# Bioelectric regulation of innate immune system function in regenerating and intact *Xenopus laevis*

**DOI:** 10.1038/s41536-017-0019-y

**Published:** 2017-05-26

**Authors:** Jean-François Paré, Christopher J. Martyniuk, Michael Levin

**Affiliations:** 1 Biology Department, and Allen Discovery Center at Tufts, Tufts University, Medford, MA USA; 2 Center for Environmental and Human Toxicology and Department of Physiological Sciences, University of Florida Genetics Institute, College of Veterinary Medicine, University of Florida, Gainesville, FL USA

## Abstract

Two key inputs that regulate regeneration are the function of the immune system, and spatial gradients of transmembrane potential (*V*_mem_). Endogenous bioelectric signaling in somatic tissues during regenerative patterning is beginning to be understood, but its role in the context of immune response has never been investigated. Here, we show that *V*_mem_ levels modulate innate immunity activity in *Xenopus laevis* embryos. We developed an assay in which *X. laevis* embryos are infected with a uropathogenic microorganism, in the presence or absence of reagents that modify *V*_mem_, prior to the ontogenesis of the adaptive immune system. General depolarization of the organism’s *V*_mem_ by pharmacological or molecular genetic (ion channel misexpression) methods increased resistance to infection, while hyperpolarization made the embryos more susceptible to death by infection. Hyperpolarized specimens harbored a higher load of infectious microorganisms when compared to controls. We identified two mechanisms by which *V*_mem_ mediates immune function: serotonergic signaling involving melanocytes and an increase in the number of primitive myeloid cells. Bioinformatics analysis of genes whose transcription is altered by depolarization revealed a number of immune system targets consistent with mammalian data. Remarkably, amputation of the tail bud potentiates systemic resistance to infection by increasing the number of peripheral myeloid cells, revealing an interplay of regenerative response, innate immunity, and bioelectric regulation. Our study identifies bioelectricity as a new mechanism by which innate immune response can be regulated in the context of infection or regeneration. *V*_mem_ modulation using drugs already approved for human use could be exploited to improve resistance to infections in clinical settings.

## Sparking immunity: Bioelectric signaling modulates immunity and regeneration

Bioelectrical signaling in the African clawed frog modulates both resistance to infection and tail regeneration. Michael Levin at Tufts University in Massachusetts, USA, and colleagues have used genetic technologies and drug treatments to manipulate the bioelectrical properties of tissues in frog embryos. Reducing the electric gradient between the inside and outside of cells (depolarization) increased the embryos’ survival rate to bacterial infection, whereas increasing the resting potential (hyperpolarization) had the opposite effect. The authors found that serotonergic signaling and an increase in the number of myeloid cells underpin depolarization-induced immunity. Interestingly, embryos undergoing tail regeneration, which triggers depolarization, also showed increased resistance to infection. The role of bioelectrical signaling in both regeneration and immune response suggests that ion channel drugs, many already in medical use, could be useful for treating infection and severe physical injuries.

## Introduction

The vertebrate immune system is divided into two categories: innate and adaptive.^1^ The former provides the first line of defense against pathogens via the actions of surface barriers, secreted antimicrobial peptides, and a subset of blood cell types. The latter is mediated by B and T lymphocytes and relies on the memory of previous exposure to the targeted pathogenic agent. Current vaccination strategies rely on adaptive immunity to create a specific memory that will stimulate immediate immune reaction upon future exposure to the pathogen and as a result, facilitate its elimination. However, with the geographic migration of subtropical diseases, emergence of new pathogenic agents (i.e., exposure to pathogens to which no adaptive memory has developed), clinical interests towards improving immune defenses of lymphocyte-deficient patients, and the need to prevent infection in battlefield and other traumatic injuries, the development of new methods to modulate innate immunity is of utmost importance.

Crucially, the immune system is relevant not only in the context of infection, but is also a major contributor to regenerative response. In general, evolutionary advances in immune defense, especially the development of a fully functional adaptive system, inversely correlate with regenerative capacity.^2, 3^ The role of immune response in regenerative events is an exciting emerging field.^4–6^ Thus, the incompletely developed immune system of embryos provides a powerful model to study relationships between regeneration and innate immune response to infection.^5, 7^ Modulating the pathways involved in embryonic regeneration could lead to potential new avenues for improving innate immune response.

Multiple strategies have been pursued to modulate the effectiveness of innate immunity, including genetic modifications of the host or chemical treatment of blood cells other than lymphocytes.^8–10^ Here, we explore the role of the host’s bioelectric properties and regenerative response in modulating the effectiveness of the innate immune response. Numerous studies have shown that all cells (not just excitable nerve and muscle) possess endogenous plasma membrane voltage gradients (*V*_mem_) that are generated by the ion channels and pumps they express. *V*_mem_ acts as a crucial regulator of cell differentiation, proliferation, apoptosis, migration, activation, neoplasia, and polarization. Furthermore, changes in the spatial distribution of specific *V*_mem_ levels across tissues regulates various organ-level functions, including developmental organ patterning and regeneration.^11–17^ We, therefore, hypothesized that the host’s bioelectric status could modulate the effectiveness of the innate immune response.

To test this hypothesis, we exploited the high amenability of *Xenopus laevis* embryos to biophysical and molecular-genetic perturbations,^7, 18, 19^ and the high level of conservation between amphibian and mammalian immunity. A significant advantage of this model organism with respect to the current study is that during *X. laevis* development, there are no detectable mature B or T cells until 12 days post-fertilization, providing a significant window of investigation in which innate immunity is the only defense the organism has against pathogens.^7, 20^ Moreover, *X. laevis* embryos constitute a robust model for regeneration,^21, 22^ allowing us to investigate how the innate immune mechanisms recruited by the regenerative response^23, 24^ are affecting the innate immune response to infection.

In this study, we used pharmacological and genetic methods to modulate the bioelectric properties of *X. laevis* embryo tissues after infection with bacteria that expressed the green fluorescent protein (GFP). We found that *V*_mem_ depolarization of the host increases its resistance to infection and that its hyperpolarization had the opposite effect. This effect is mediated by a serotonergic signaling pathway at the organismal level and involves migration of embryonic myeloid cells. We also show that *X. laevis* embryos undergoing tail regeneration/repair show increased resistance to infection. This suggests that bioelectric modulators, including many small molecule drugs already approved for human use, as well as signaling molecules activated by regeneration pathways, represent promising new approaches for manipulating innate immunity responses following exposure to new pathogens.

## Results

### Uropathogenic *E. coli* strains colonize *X. laevis* embryos

**Fig. 1:**
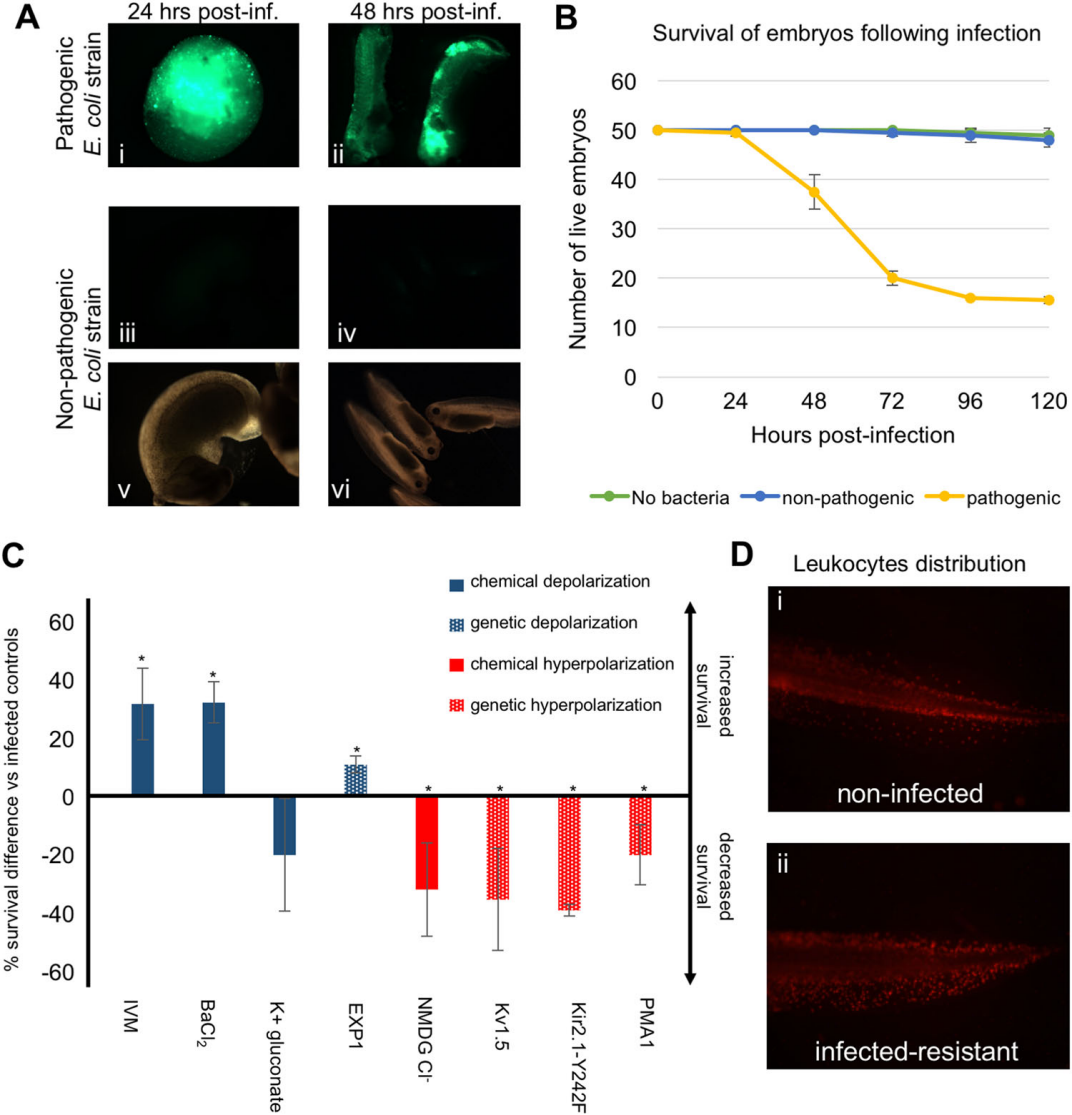
Uropathogenic *E. coli* can produce systemic infection of gastrula-stage *X. laevis* embryos, whose *V*_mem_ modulates immune response to infection. **a** Widely distributed uropathogenic *E. coli*-derived-GFP fluorescence is detected under fluorescence microscopy in embryos infected 24 h (panel i) or 48 h (panel ii) before the image was captured. In contrast, the non-pathogenic K12 *E. coli* lab strain harboring the same GFP reporter fails to colonize the embryos after injection (fluorescent imaging in panels i–iv, bright field imaging in panels v–vi). **b** Comparative survival curves of embryos injected with vehicle solution (no bacteria, *green line*), non-pathogenic *E. coli* (*blue line*), or uropathogenic *E. coli* (*yellow line*). Error bars represent the standard deviation from three biological replicates. Embryos showing no motion after poking or being in a state of lysis were counted as dead. **c** Embryo’s *V*_mem_ modulates immune response to infection. General embryo depolarization increases the rate of survival following infection while general embryo hyperpolarization decreases post-infection survival. For chemical polarizations, embryos were kept in normal conditions until after infection (late gastrula stage), at which point the specified compounds were added to their media. For genetic modifications of polarity, one-cell embryos were injected with mRNAs coding for the specified transmembrane channels, incubated until they reach the gastrula stage, and infected with uropathogenic *E. coli*. Surviving embryos were counted 4 days after infection (chemical depolarization conditions in solid *blue columns*, chemical hyperpolarization condition in *solid red column*; genetic depolarization condition in *dotted blue column*, genetic hyperpolarization conditions in *dotted red columns*). Error bars represent the standard deviation from at least three biological replicates. *denotes *p* < 0.05. *IVM* ivermectin, *NMDG N*-methyl-d-glucamine. **d** Resistant embryos show peripheral mobilization of leukocytes. Fluorescence detection of leukocytes with the specific XL2 antibody in embryos surviving 4 days after infection. Panel i shows a non-infected embryo while panel ii shows an infected one

Our initial goal was to identify pathogenic bacteria that would infect *X. laevis* embryos and could be easily detected and quantified using fluorescence. Up to developmental stage 48, innate defenses are the only immune resistance active in these transparent embryos, making *X. laevis* embryos an ideal model for studying modulation of innate immunity.^7, 20^ We selected a subset of uropathogenic *E. coli* strains (generously provided by Dr.Matthew A. Mulvey, University of Utah^25^) based on their ability to infect evolutionarily distant model species (adult mouse and zebrafish embryos) and eliciting immune responses. Moreover, these strains are traceable due to their harboring of a plasmid encoding an unstable form of the green fluorescent protein (GFP-LVA mutant) that allows the experimenters to visualize the progression of the infection, and whose very short half-life circumvents fluorescent noise left by dead bacteria. We first tested the potential of the bacteria to infect embryos at stages 8–9 (blastula) or 11–12 (gastrula). No propagation of the non-pathogenic *E. coli* strain (K12 lab strain) was detected in embryos infected with it at either stage, while the pathogenic strains showed clear propagation throughout many anatomical regions in the majority of infected embryos (Fig. 1a). These data show that the uropathogenic strains have the capacity to colonize *X. laevis* embryos at early stages of embryonic development.

We then determined the survival rate of embryos daily for 6 days after infection. When compared to non-infected embryos, no lethality was found among the embryos infected with the non-pathogenic strain. However, only 30–50% of infected embryos survived when the uropathogenic strains were used, with most of the lethality occurring between 24 and 72 h following infection (example shown in Fig. 1b). Thus, since the adaptive immune is not yet developed at these developmental stages, we established that survival rates of *X. laevis* embryos after infection with human-relevant bacteria could be used as a proxy for the degree of activation of the innate immune system.

### Chemical modulation of the bioelectric state of the embryo affects resistance to infection

Many physiological and developmental processes have been shown to be regulated by bioelectrical signaling.^14, 26–30^ We, therefore, investigated whether the organism’s bioelectric state could modulate innate immunity activity in vivo. It is important to note that in this study, the terms bioelectric depolarization or hyperpolarization are used in the sense of electrophysiology (referring to plasma membrane resting potential), not “polarization” in the sense of cell activation by chemical chemoattractants or cytokines as described in immunology literature. First, we targeted chloride channels with IVM, which selectively opens glycine receptor chloride (GlyCl) channels in the cell membrane. Since the concentration of Cl^−^ is higher in the intracellular environment (40–60 mM) than in the extracellular one (10 mM), treating the cell with IVM leads to its depolarization, as the negatively charged chloride ions exit the cell through the open GlyCl channels. This strategy has recently been used to efficiently depolarize host tissues and exploited to test the role of tissue resting potentials in guiding innervation,^31^ muscle patterning,^32^ and metastasis.^33, 34^ Treatment of the embryos with 1 μM IVM, a dose that does not inhibit bacterial growth,^35, 36^ one hour after infection led to a significant increase in the ratio of surviving embryos (31.7 +/− 12.3%; Fig. 1c) when compared with untreated infected ones. Interestingly, by using a medium with higher concentrations of Cl^−^ ions (70 mM NMDG-chloride), which leads to hyperpolarization,^31, 34^ we observed the inverse effect: there was a significant decrease in the survival ratio (−32.0 +/− 16.0%; Fig. 1c).

To determine whether this effect was chloride-specific or due to depolarization of *V*_mem_ *per se*, we tested another depolarizing compound, barium chloride, which is a well-known potassium channel blocker.^37–39^ We found that it had an effect similar to IVM, significantly increasing the survival ratio (32.3 +/− 7.0%; Fig. 1c). This suggests that the previously observed effect on infection-resistance was not specific to one compound or a type of ion, but rather due to the reagents’ depolarizing action. Membrane voltage readings on embryos treated with barium chloride showed increased depolarization when compared with untreated embryos (Supplementary Fig. 1), indicating the effectiveness of the treatment in depolarizing embryos. We also assayed a medium that was highly concentrated in potassium ions (60 mM), which depolarizes embryos by preventing the exit of K^+^ ions through potassium channels, by supplementing with potassium gluconate (as in prior studies).^40–42^ Potassium gluconate (K-gluc) must be used in such bioelectricity studies instead of the commonly-used KCl because the excess chloride ions can confound the results. Surprisingly, no significant increase in survival ratios was observed (−20 +/− 19.3%; Fig. 1c). We hypothesized that this was due to gluconate serving as a nutritious agent for *E. coli*: if true, any increase in the host resistance to infection would be masked by an increase in bacterial proliferation independently from the host’s bioelectric state. To test this hypothesis, we grew the pathogenic strain on its own in suspension in media supplemented with K-gluc and observed a pronounced increase in bacterial proliferation compared to controls (Supplementary Fig. 2). In addition, we showed that IVM and barium chloride do not have any negative effect on bacteria growth. Taken together, our data suggest that depolarization of host tissue leads to an increase in its resistance to infection, while hyperpolarization decreases it.

### Genetic modulation of the bioelectric state of the embryo affects resistance to infection

Interestingly, bacteria also utilize bioelectric signaling at their membranes.^43, 44^ To confirm that the aforementioned effects were not simply due to the compounds’ impact on bacteria survival/proliferation (or their own bioelectric state), we injected fertilized eggs with mRNAs encoding ion channels that reliably lead to depolarization or hyperpolarization in *Xenopus* ^45^—this specifically targets the resting potential of host cells, by expressing these channel proteins in the larval tissues. We first used the EXP1 channel, a constitutively conductive cation channel from the nematode *C. elegans* that acts as a depolarizing agent under these conditions.^46^ Embryos were injected at the one-cell stage with the highest dose of mRNA that did not cause an increase in spontaneous lethality in non-infected embryos and avoided developmental defects or toxicity. We found that the ratio of survival to infection was significantly increased by 11.0 +/− 2.8% in *EXP1*-injected embryos (Fig. 1c), reinforcing our previous finding that the observed effects with chemical depolarization were not simply caused by the compounds’ direct action on bacteria and were not due to off-target effects of drug compounds, but rather were a specific consequence of bioelectric potential change in the host cells.

We then used forced expression of hyperpolarizing ion translocators to determine the effect of genetic hyperpolarization of the host on resistance to infection. Overexpression of two hyperpolarizing potassium channels, Kv1.5 and an overactive Kir2.1 mutant,^47–49^ led to respective significant decreases of 35.3 +/− 17.4 and 39.0 +/− 2.0% in survival after infection (Fig. 1c), confirming at the molecular-genetic level the outcome previously obtained with chemical hyperpolarization. We also hyperpolarized the embryos by injecting the *PMA1.2* mRNA, which encodes a plasma membrane-localized proton pump previously used to hyperpolarize *Xenopus* cells.^50, 51^ Similar to the effects of hyperpolarizing potassium channels, we observed a significant decrease of 20.0 +/− 10.2% in survival after infection, suggesting that the effect of genetic hyperpolarization was due to the general hyperpolarized state of the embryos, and not specific effects of individual cation types (Fig. 1c).

Taken together, our data from the chemical and genetic modification of the embryos bioelectric state using multiple different ion families, pharmacological agents, and genetic translocators, indicate that depolarization of the host increases resistance to infection, while hyperpolarization decreases it.

### Mobilization of leukocytes in survivors is not affected by their bioelectric state

In order to probe the mechanism(s) by which the increase/decrease in infection-resistance linked to the host’s bioelectric state, we studied the mobilization of leukocytes (a key mediator of innate immunity) following infection in the multiple bioelectric states described above. We followed leukocyte localization within the embryo by immunofluorescence using XL2, an antibody that specifically recognizes *Xenopus* embryonic leukocytes.^52^ We first found that embryos surviving infection 96 h after inoculation were showing peripheral mobilization of embryonic leukocytes: the non-infected controls had XL2-labeled leukocytes concentrated in the middle part of the body (enriched along the borders of the trunk tissues), while the resistant embryos showed a more peripheral distribution of these cells into the fin (Fig. 1d), indicating an infection-triggered migration. This clearly demonstrated that the survivors were exposed to the infectious agents and not simply surviving as a result of experimental inefficiency in the infection protocol (as all specimens were exposed).

Supplementary Figure 3A shows the mobilization of XL2-labeled cells in the periphery of an infected tadpole 4 days post-infection: the density of labeled leukocytes was higher in the ventral fin (right panel) when compared to the non-infected tadpole (left panel). We quantified the density of XL2-labeled cells in the post-anal portion of the ventral fin of non-infected and infected tadpoles for all the previously studied bioelectric states (via the automated counting of XL2 spots in single-color images using ImageJ). In non-infected embryos, only chemical hyperpolarization with high extracellular Cl^−^ concentrations led to a significant increase in relative leukocyte mobilization (Supplementary Fig. 3B, panel iii). Following infection, all conditions showed significant increases in relative leukocytes concentrations in the ventral fin when compared to non-infected tadpoles, but no infected group in a modified bioelectric state showed a significant difference with infected controls (Supplementary Fig. 3B, panels i–iv). This suggests that modification of the organism’s bioelectric state does not result in significant variations in leukocyte mobilization.

### Chemical hyperpolarization increases bacterial load in the host

To gain additional insight into the events leading up to the effect of *V*_mem_ on animal survival end points, we characterized bacterial load profiles at intermediate time-points. To quantify bacteria at various times following infection, we adapted a protocol for the quantification of GFP (as the bacteria we are using harbor a GFP-expressing plasmid) activity in embryo lysates.^25^ Preliminary measurements indicated that the level of infection was sufficient starting at 48 h post-infection to be detectable by spectrofluorometry in non-denaturing embryo lysates (data not shown). We compared the levels of infection at the 72 h post-infection time point (Supplementary Fig. 4) and found that, when compared to controls, the average, median, and third quartile values were all decreased in the two depolarizing conditions leading to an increased survival rate. However, these differences were not statistically significant (*p* = 0.195 and 0.127 for IVM and barium chloride treatments, respectively).

Interestingly, the hyperpolarizing condition (high chloride) led to a significant increase in bacteria-derived GFP activity (*p* = 0.008). We also noted that the presence of potassium gluconate leads to a significant increase in bacteria-derived GFP signal (*p* = 0.019), which correlates with this compound not increasing the survival rate of infected embryos. The lower averages and quartiles observed in the IVM and barium chloride conditions are due to the higher number of embryos showing no detectable level of bacteria-derived fluorescence: these embryos will eventually survive and, although some had visible GFP levels 24 h post-infection, the levels were too low to be detected by spectrofluorometry on whole embryo lysates. Given that all embryos showing visible GFP activity at 48 and 72 h die (Supplementary Fig. 5), we suggest that the critical time for triggering an immune reaction sufficient to eliminate and resist the infection occurs within the first 24 h following infection and that, once a certain bacterial threshold is reached within the host, the progression of infection becomes irreversible and its rate of progression is impacted by the host’s bioelectric status.

### Depolarization-induced increase in survival is mediated by a serotonergic pathway

**Fig. 2:**
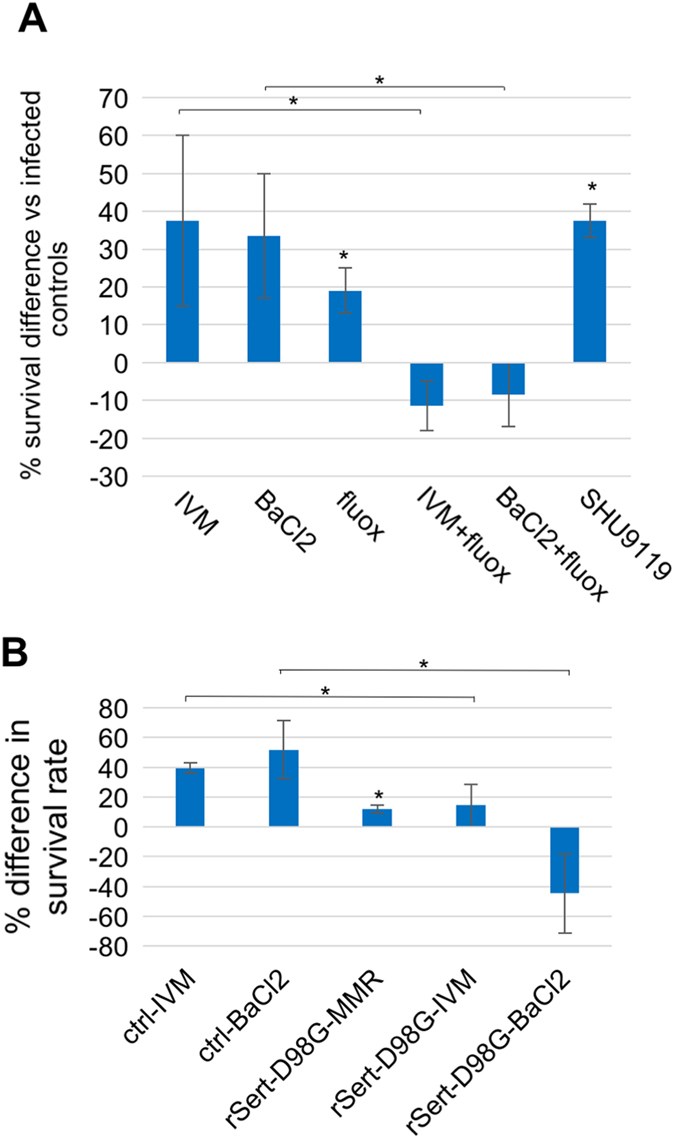
Increased resistance to infection following chemical depolarization is mediated through a serotonergic signaling pathway. **a** Following infection, embryos were immediately incubated with the specified compounds. **b** For genetic inhibition of serotonergic signaling, a dominant-negative rat serotonin transporter mRNA encoding a mutant (rSert-D98G) was injected into one-cell embryos, which were grown to the gastrula stage, and infected with uropathogenic *E. coli*. Surviving embryos were counted 4 days after infection. Error bars represent the standard deviation from two biological replicates. *denotes *p* < 0.05. *IVM* ivermectin, *fluox* fluoxetine

Previous studies have shown that bioelectric changes are often transduced into downstream pathways and changes in cell behavior by voltage regulation of serotonergic signaling.^31, 33, 34, 53–55^ We asked whether a similar signaling pathway was involved in the increased resistance of depolarized embryos to infection, since IVM treatment leads to both hyperpigmentation and increased survival following infection. We exploited a suppression/rescue strategy that targets the serotonin transporter SERT, a key player in the regulation of serotonin movement by resting potential. We repeated the infection experiments with embryos depolarized using IVM or barium chloride, and observed a similar average increase in survival (37.5 +/− 22.5% and 33.5 +/− 16.5% for IVM and barium chloride, respectively; Fig. 2a) as reported above. When we combined these treatments with fluoxetine, a specific inhibitor of serotonin transport,^56, 57^ the depolarization-induced increases in survival were nullified, suggesting the involvement of a serotonergic pathway in bioelectrically-induced resistance to infection. Similarly, when treated with SHU9119, an MSH agonist leading to a hyperpigmented phenotype acting downstream from serotonergic signaling following treatment with IVM,^33^ we likewise observed a significant increase in resistance to infection (37.5 +/− 4.5%; Fig. 2a), indicating that hyperpigmentation was sufficient for this increase, and/or that additional pro-resolution functions of MSH signaling^58, 59^ directly affected immune system functions. Taken together, these results identify signaling pathways involving serotonin and/or MSH in the resistance to infection.

We then molecularly validated the role serotonergic signaling plays in depolarization-induced increased resistance to infection by substituting fluoxetine treatment with the injection of rSert-D98G, an mRNA encoding a dominant negative mutant form of the rat serotonin transporter.^55, 60^ As observed with fluoxetine, expression of the dominant negative serotonin transporter significantly reduced (for ivermectin treatment) or nullified (for BaCl_2_ treatment) the depolarization-induced increase in the resistance to infection (Fig. 2b). Together with the results obtained with fluoxetine treatments, these data confirm that a signaling pathway mediated by serotonin modulates depolarization-induced resistance to infection.

### Melanocyte-derived compounds increase survival following infection

**Fig. 3:**
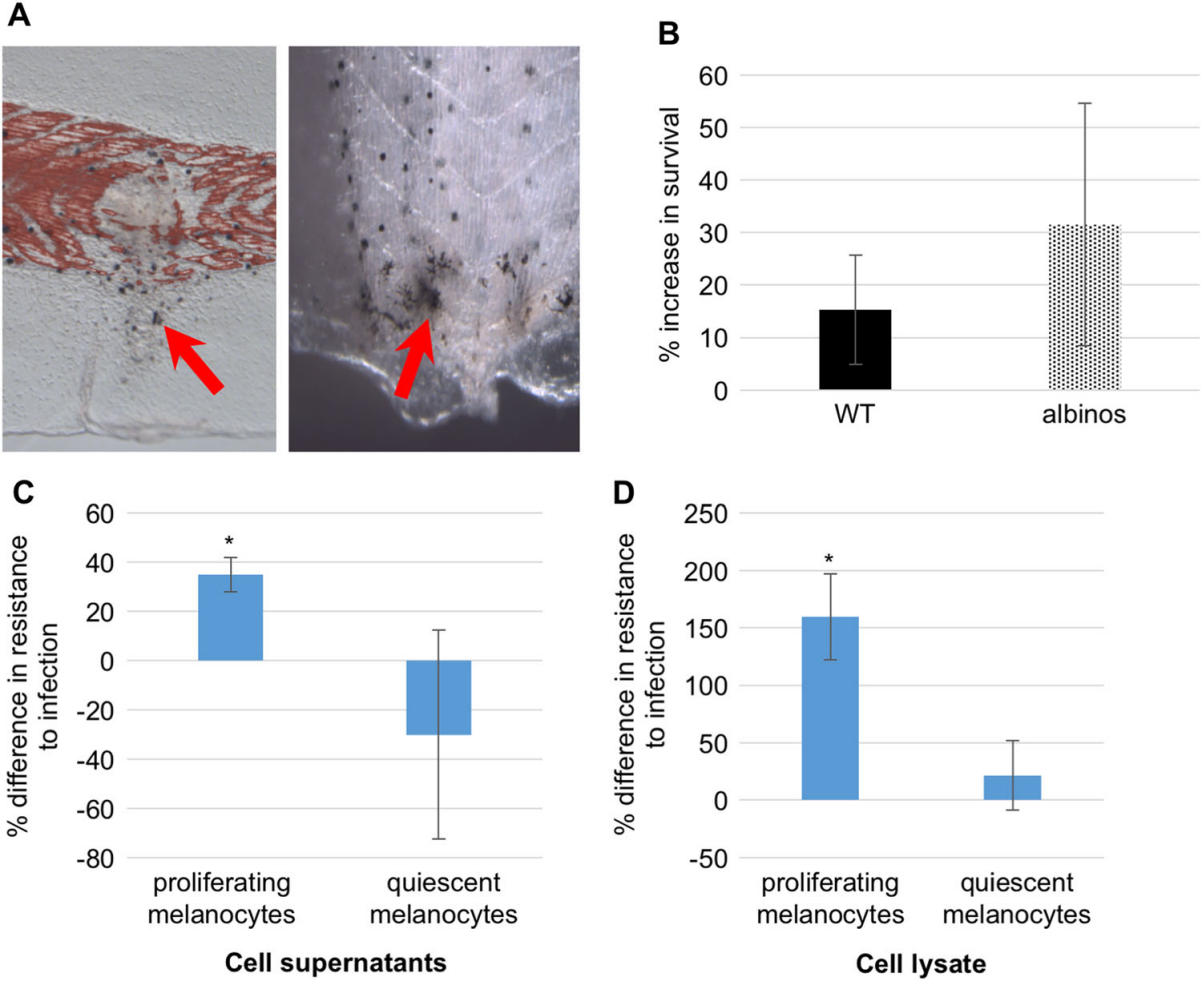
Melanocyte-derived factors increase resistance to infection. **a** Melanocytes migrate and fragment to injury sites. **b** Albino embryos’ resistance to infection keeps being increased by exposure to ivermectin. Infected embryos were exposed to ivermectin for 24 h following infection and the survival ratios were calculated 4 days post-infection. **c** Percentage increase in survival to infection following bacterial exposure to cell supernatants from proliferating or quiescent human neonatal melanocytes (HEMn). Error bars represent the standard deviation from three biological replicates. **d** Percentage increase in survival to infection following bacterial exposure to non-denaturing cell lysates from proliferating or quiescent human neonatal melanocytes (HEMn). Each column represents the compilation of three independent experiments within which each experiment used embryos from a single fertilization. *denotes *p* < 0.05

The increase in resistance to infection observed following treatment with an MSH agonist (SHU9119; Fig. 2a) suggests that melanocytes could have an active role in mediating innate immunity. This possibility is further supported by the fact that melanocytes have been shown in zebrafish to migrate to wound sites and then fragment releasing their contents.^61^ We found this to also be the case in *Xenopus*, in muscle punctures and tail amputations (Fig. 3a). To test the role of melanocytes in immune response, we treated infected albino embryos, whose melanocytes do not synthesize melanin, with IVM and quantified the surviving embryos. We found that the IVM-induced increase in resistance to infection was still present in albinos (31.5 +/− 23.1 % in albinos vs. 15.3 +/− 10.4 % in wild-type; Fig. 3b), suggesting that the melanocyte-dependent effect observed was not dependent on the synthesis/secretion of melanin.

We then tested the effect of factors secreted by melanocytes as well as of their intracellular content on sensitivity to infection. Two subgroups of cultured human melanocytes were evaluated: low density, in which the melanocytes are proliferating and do not show pigmentation, and high density, in which the melanocytes are confluent, quiescent, and pigmented. We found that both the supernatant and cell lysate from actively proliferating melanocytes significantly (*p* < 0.05) increased the resistance to infection compared to control infected animals (35.0 +/− 7.0% and 159.5 +/− 37.5% for supernatant and lysate, respectively; Figs. 3c–d), while no significant difference was observed with extracts derived from quiescent, melanin-producing melanocytes (−30.0 +/− 42.4% and −21.5 +/− 30.4% for supernatant and lysate, respectively; Figs. 3c–d). These data suggest that non-quiescent melanocytes secrete and harbor factors other than melanin that modulate the innate immune response to increase survival following infection.

### Immunity-related transcriptional events downstream of IVM-induced depolarization

**Fig. 4:**
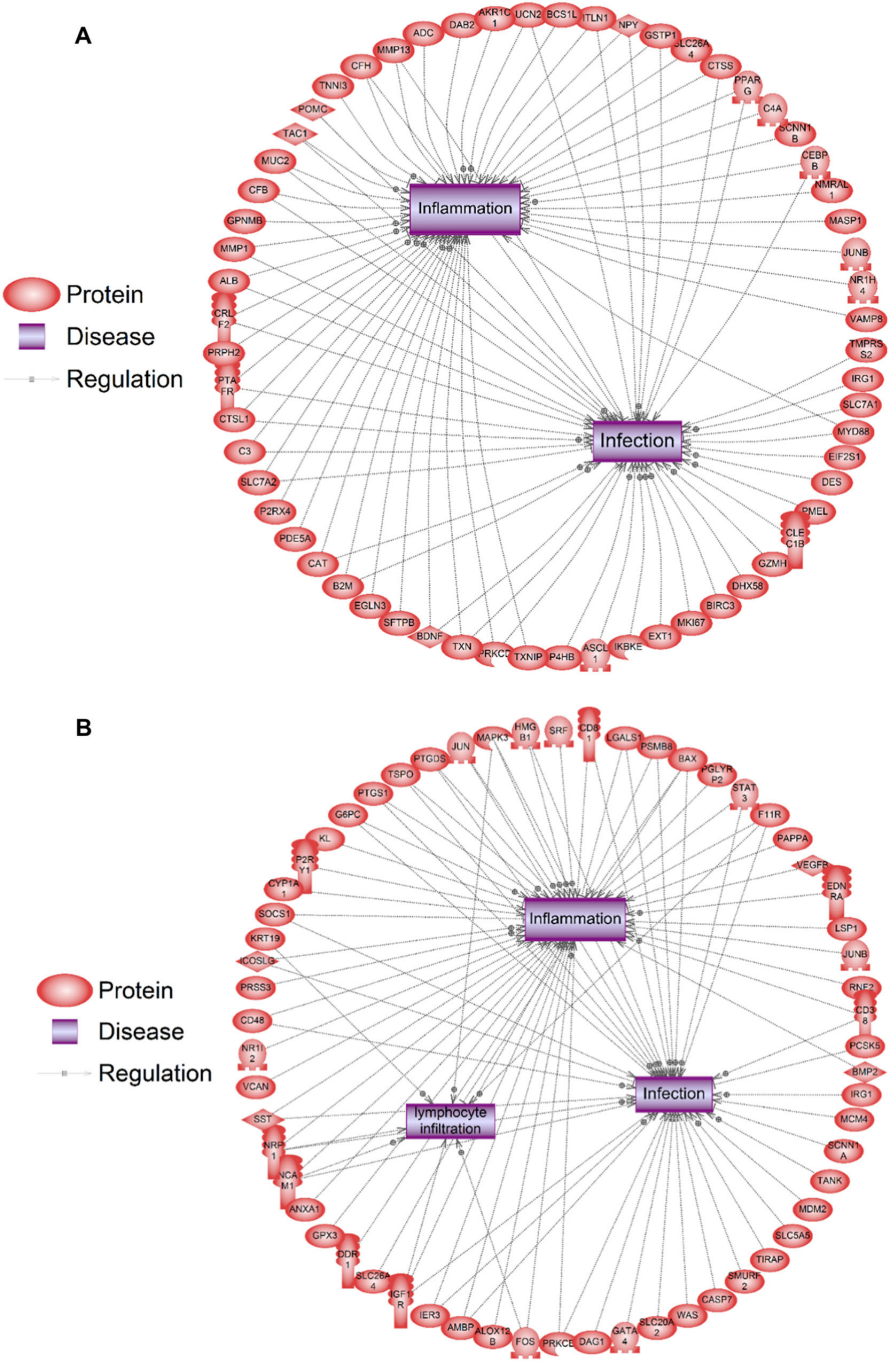
Human homologs of immune genes differentially expressed at stage 45 after depolarization with ivermectin **(a)** or glycine receptor overexpression **(b)**. Proteins are *red shapes*, diseases are *purple boxes*, stimulatory regulatory events are indicated by an *arrow* and a *plus sign* on the relationship line, inhibitory regulatory events are indicated by a *blunt line*, and arrows without any sign indicate direct binding of proteins

Since depolarization of instructor cells by IVM led to increased resistance to infection, we performed a transcriptomic analysis on stage 45 tadpoles to identify likely transcriptional mediators of this effect. Stage 45 was chosen due to its advanced stage of development, which yielded a higher number of innate immunity-related candidates when compared to earlier stages (analyzing at st. 15, we found only 19 transcripts to have been altered, with no known links to immune system function; data not shown). We compared embryos treated with IVM with untreated embryos, as described in (ref. 33). This analysis identified 517 differentially expressed probes following IVM treatment (Appendix 1). Several of these genes correspond to human homologs involved in immune pathways and were associated with immune-related physiological phenomena such as infection (Fig. 4a). Transcripts associated with immunity and those downregulated more than two-fold included eukaryotic translation initiation factor 2, subunit 1 alpha, cathepsin L1, and albumin while those upregulated by more than two-fold included platelet-activating factor receptor, thioredoxin, complement component 4A (Rodgers blood group), complement component 6, gene 1, cathepsin S, and immunoresponsive gene 1.

Using a second, independent transcriptome dataset in Xenopus, we characterized transcript changes that occurred via glycine receptor activation^40^ and identified 520 differentially expressed probes (Appendix 2). Despite the fact that there was only 5% overlap of common probes within each data set, processes related to inflammation and infection were also significantly represented by these transcripts (Fig. 4b). Network enrichment analysis for expression targets revealed that transcripts regulated by glycine receptor agonism are regulated by molecules such as IL1B, IL11, NF-kappaB, interleukins, and tumor necrosis factor. Moreover, gene network analysis for cell processes with glycine receptor activation^40^ revealed that many differentially expressed probes were related to inflammation, infection, lymphocyte infiltration, and wound healing. Transcripts in this network included solute carrier family 20 (phosphate transporter), member 2, immunoresponsive 1 homolog (mouse), proteasome (prosome, macropain) subunit, beta type, 8 (large multifunctional peptidase 7), and annexin A1 were upregulated more than 20-fold while protease, serine, 3, CD48 molecule, solute carrier family 26, member 4, and solute carrier family 5 (sodium iodide symporter), member 5 were downregulated more than 20-fold. Other genes in this network associated with immunity and infection were toll-interleukin 1 receptor (TIR) domain containing adaptor protein, CD38 molecule, CD48 molecule, CD81 molecule, inducible T-cell co-stimulator ligand. Both these transcriptomic studies support the idea that improved resistance to infection after depolarization may be mediated in part through the differential expression of transcripts, conserved between frog and mammals, whose products play a role in the immune system.

### Depolarization increases population of extracellular matrix remodeling macrophages

**Fig. 5:**
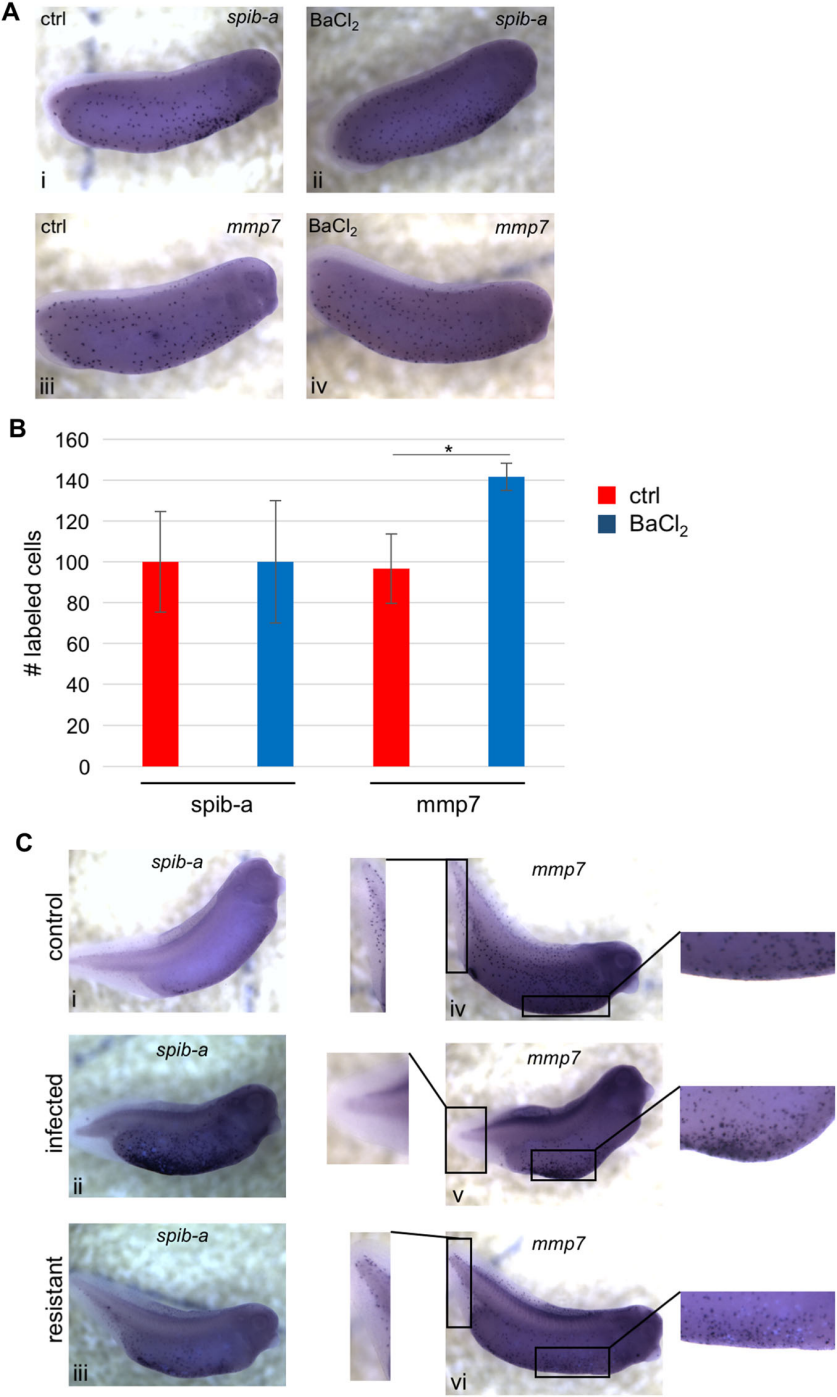
Barium chloride-induced depolarization affects primitive myeloid cells distribution. **a** Visualization of *spib-a* and *mmp7* expression by in situ hybridization at stage 26 in control (i, iii) and barium chloride-treated (ii, iv) conditions. **b** Quantification of *spib-a* and *mmp7*-positive cells in control and barium chloride-treated conditions reveals that depolarization by barium chloride significantly increases the number of *mmp7*-expressing cells at stage 26. Error bars represent the standard deviation from five biological replicates. *denotes *p* < 0.05. **c** Visualization of *spib-a* and *mmp7* expression by in situ hybridization at stage 33 in control (i, iv), infected (ii, v), and resistant (iii, vi) embryos

In order to identify additional cell-level mechanisms that mediate the increase in resistance observed following barium chloride-mediated depolarization, we examined the expression of an early primitive myeloid marker, *spib-a*,^62^ and a gene coding for a protein mediating extracellular matrix remodeling (and, therefore, allowing migration) by macrophages, *mmp7* (ref. 63). Shortly after hatching (stage 26), the number of *spib-a*-positive cells was similar in animals from all conditions, but the number of *mmp7*-positive cells was significantly increased in barium chloride-treated specimen (283 +/− 13 vs. 193 +/− 34 per specimen for barium chloride and control conditions, respectively; Figs. 5a–b). These data suggest that the depolarizing treatment does not affect primitive myeloid cells’ commitment, but acts to increase the proportion of committed myeloid cells acquiring the capacity to migrate throughout the embryos.

At stages 33/34, the majority of *spib-a*-expressing cells localized within the yolk region and a sizable portion resided in the posterior fin region (Fig. 5c(i)). In embryos showing detectable levels of bacteria-derived GFP activity (qualified as infected), *spib-a* expression intensified in the ventral yolk region and was absent from the periphery (Fig. 5c(ii)), contrary to resistant embryos (no detectable GFP) in which *spib-a* expression was still present in the periphery and closer to control levels in the yolk region (Fig. 5c(iii)). For *mmp7*, expression was detected in cells distributed all over the embryo (Fig. 5c(iv)), becoming concentrated in the ventral yolk region in infected specimens (Fig. 5c(v)), while resistant embryos showed a pattern of expression closer to that of controls (Fig. 5c(vi)). These data indicate that the infection status influences the localization of both *spib-a-* and *mmp7*-positive myeloid cells, and suggests that their presence at the embryo periphery is crucial for survival after infection. The increase in *mmp7*-positive cells (and, therefore, migratory immune cells) shown in embryos depolarized with barium chloride could explain the observed increase in infection-resistance.

### Tail amputation increases survival to infection

**Fig. 6:**
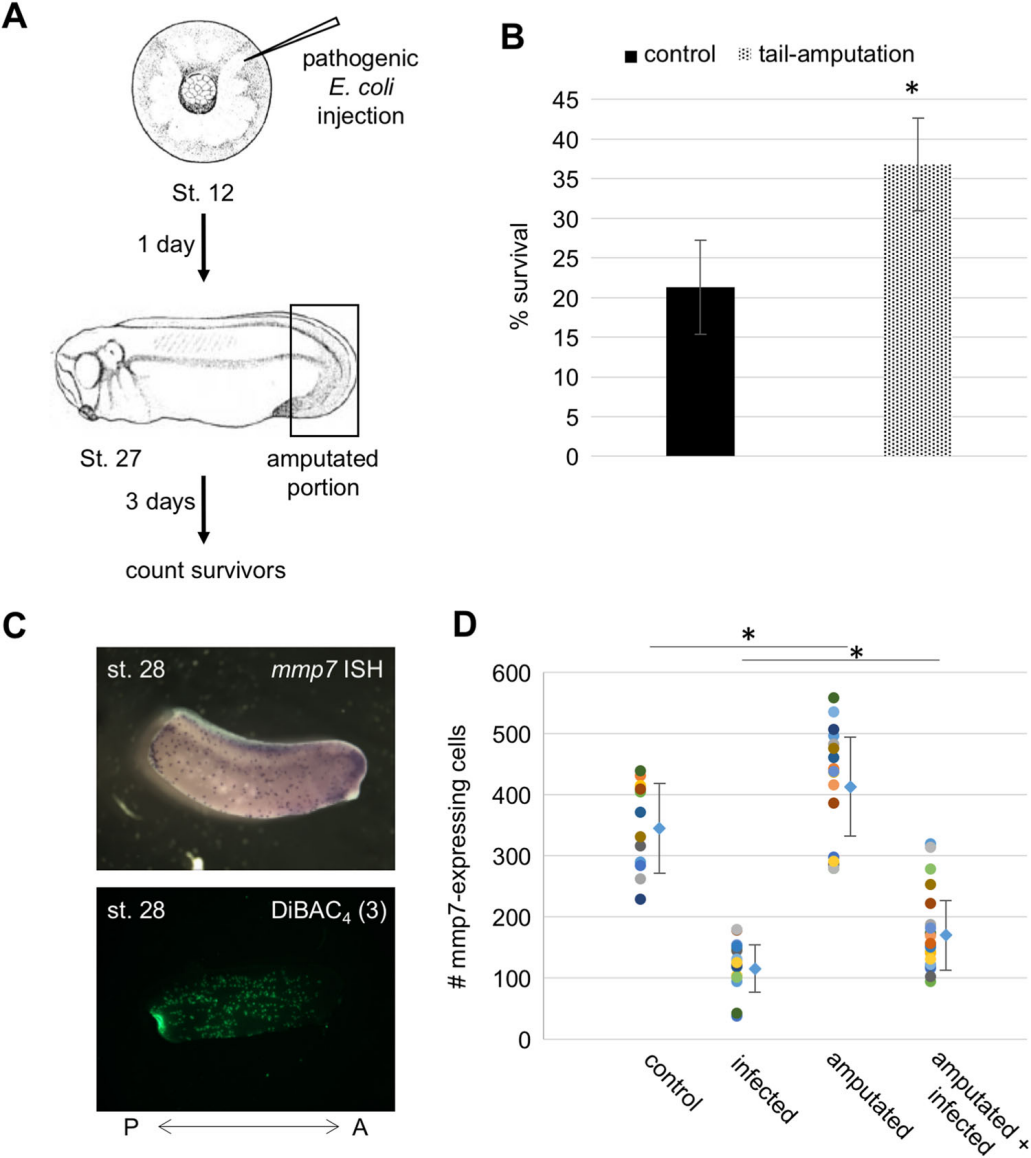
Tail amputation increases resistance to infection. **a** Embryos at NF stage 12 (gastrula) were infected with uropathogenic *E. coli* and, after reaching NF stage 27 (post-hatching), were amputated from the posterior fifth of the body. The number of survivors was quantified 3 days later (4 days post-infection) and compared with non-amputated infected embryos. **b** Comparison of survival to infection percentages between non-amputated (*plain column*) and amputated (*dotted column*) embryos reveals an augmentation of survival by tail amputation and subsequent regeneration. Each column represents three independent assays. **c** Visualization of *mmp7*-expressing myeloid cells by in situ hybridization following amputation (*top panel*) reveals increased posterior concentration of primitive myeloid cells, and detection of depolarized cells by staining with DiBAC_4_(3) voltage dye (*lower panel*) reveals a cluster of highly depolarized cells at the posterior extremity of the embryo after tail amputation. **d** Quantification of *mmp7*-expressing cells following infection and/or amputation at NF stage 28 reveals significant increases in *mmp7*-expressing cells consequent to tail amputation. *denotes *p* < 0.05

Tail amputation has been shown to depolarize the *X. laevis* embryos at the amputation site,^21, 42^ and regeneration is now known to involve the recruitment of innate immunity mediators to the site of injury in order to form a blastema and lead to successful development of the ablated tissues.^23, 24^ Bioelectric signaling is important in both regenerative response^64–66^ and immune modulation, but no prior studies have examined bioelectrics, immunity, and regeneration in the same context. We thus investigated the effect of tailbud amputation of the host on survival following infection. When tailbuds from infected embryos were amputated (as schematized in Fig. 6a), we found that their survival percentage was significantly increased (21.3 +/− 5.9% vs. 36.8 +/− 5.8% for non-amputated and tail-amputated, respectively; Fig. 6b).

We then determined the localization of *mmp7*-expressing cells by in situ hybridization of amputated embryos and found that some specimens mobilized a subpopulation a myeloid cells at the site of injury as soon as two hours after amputation (Fig. 6c, top panel), a localization that correlates with that of a foci of depolarized cells at the site of injury as visualized with a voltage dye (Fig. 6c, lower panel). Quantification of *mmp7*-expressing cells in whole populations of embryos amputated and/or infected showed that, at stage 28 (2 h after amputation), infection resulted in a decreased amount of *mmp7*-expressing myeloid cells. However, tail amputation led to a significant increase in *mmp7*-expressing cells in both non-infected and infected specimens when compared to their non-amputated counterpart (416.3 +/− 95.5 vs. 348.8 +/− 72.5, *p* = 0.049 for non-infected amputated and non-amputated, respectively; 177.0 +/− 67.2 vs. 123.4 +/− 38.0, *p* = 0.004 for infected amputated vs. non-amputated, respectively; Fig. 6d). This shows that an intervention that depolarizes a portion at the periphery of the embryo^42, 67^ and leads to the recruitment of myeloid cells to the site of injury can successfully increase the resistance of embryos to infection.

## Discussion

### Establishing *Xenopus laevis* embryos as a vertebrate model for innate immunity

In this study, we demonstrated that uropathogenic strains of *E. coli* are able to colonize and infect *Xenopus laevis* embryos. Under the described method, the majority of infected embryos succumb to infection within 4 days, while the surviving minority shows peripheral mobilization of leukocytes indicative of an activated immune response. Considering functional lymphocytes only reach detectable levels later during development,^7, 20^ the observed immune response is achieved via innate immunity. Crucially, the rate of survival is modulated by the bioelectric status of the host: when chemical or genetic depolarization of the infected embryos occurs at levels that do not affect the viability of uninfected controls, resistance to infection increases, and the survival rates are higher. Conversely, forced hyperpolarization of embryos leads to the opposite effect. Most importantly, triggering regenerative pathways enhances immune response efficiency in eliminating the pathogen.

We characterized the response of animals to bioelectric modulation both, at the protein level (via serotonergic signaling) and at the transcriptional level (microarray analysis); as in other contexts, this system has both a physiological component involving signaling molecule transporters (SERT) and an mRNA component that regulates numerous target genes. These results show that *X. laevis* embryos are a robust model for studying or screening modulators of innate immune response and for characterizing the cellular and molecular components of this response, as well as their relationship to regeneration, with respect to both molecular-genetic and biophysical parameters. Moreover, the ease in obtaining large quantities of fertilized embryos and the infection method (direct injection) makes it a relatively high-throughput screening method, which could become even more effective with the future development of robotic automated injection.

### Bioelectricity as a modulator of innate immunity

Our study adds innate immunity to the list of biological systems that are endogenously regulated by bioelectrical signaling, and can be modulated by targeting *V*_mem_.^27, 68, 69^ Previous studies had shown that *V*_mem_ plays important roles in a wide range of biological phenomena, including axial patterning, wound healing, appendage regeneration, craniofacial development, eye and brain development, cell migration, and cancer.^14, 15, 70^ In the present study, chemical treatments of *Xenopus* embryos with compounds leading to general depolarization of the organism led to increased resistance to infection from uropathogenic *E. coli* strains, while hyperpolarization led to a higher susceptibility. Potassium gluconate was the exception to that rule, with an increased susceptibility to infection in embryos treated with the compound at a concentration that led to depolarization. However, we found that potassium gluconate strongly stimulated bacterial growth, which likely cancels the effect expected by the depolarizing fraction of the compound. None of the other chemical treatments (IVM, barium chloride, high chloride) had any effect on bacterial growth, showing that the observed effects were consequent to modifications in the properties of the host.

It is important to note that the use of channel misexpression confirmed the modification of immune response to be mediated by bioelectric effects on host cells, not on the bacteria directly. The weaker increase in infection resistance obtained from EXP1 depolarizing channel expression, as compared with the effects of chemical depolarization, could result from GABA sequestration by the overexpressed EXP1 channels, which would then decrease normal GABA signaling through native receptors, a known modulator of immune response.^71^

Previously-reported effects of ion channel drugs on the immune response^72^ are consistent with our data, and provide a long-sought mechanism of action. Since the reagents we employed utilized diverse ion types, it is likely that *V*_mem_ itself, and not the action of specific ions, is the signal modulating the immune response; this same ion-independence (true voltage control) has been observed in the contexts of metastatic conversion,^34^ eye induction,^73^ neural outgrowth,^31^ and brain size regulation.^17^ This is also supported by our observations that *V*_mem_ modulation by genetic methods impacted resistance to infection in the same direction as chemical modulation of *V*_mem_

When peripheral mobilization of leukocytes was measured, we found that all the embryos that resisted and survived the infection showed a significant increase in the peripheral concentration of leukocytes. However, the mobilization was not significantly different when infected controls were compared with infected depolarized/hyperpolarized embryos. This suggests that bioelectricity acts as a modulator of the initial immune response to the infection, before bacteria-derived fluorescence reaches detectable levels. There is a narrow window in which the immune response can eliminate the infectious agent; if the response is not sufficiently strong, the progression of the infection is irreversible.

Our data regarding *mmp7* expression patterns support the idea of an early immune response being a key determinant in the resistance. Control embryos harbor more *mmp7*-expressing cells in early tailbud stages, and widely distributed expression is a feature shared by all resistant embryos, suggesting that extracellular matrix remodeling mediated by *mmp7*-expressing activated macrophages, and the macrophages’ enhanced migratory activity, could be essential for a sufficient early immune response. The numbers of myeloid cells are not significantly different after depolarization with barium chloride, as shown by *spib-a* expression quantifications, which suggests that early myeloid commitment is not affected by bioelectric modulation. However, the maturation toward metalloproteinase-expressing cells is enhanced by depolarization. This observation about an increase in migrating cells having a role in depolarization-mediated increase in resistance to infection is supported by our tail amputation experiments showing that infected embryos amputated from their tail show an increase in survival to infection: innate immunity mediators, e.g., myeloid cells, are known to be mobilized to the site of injury^74, 75^ which in our case would result in increased peripheral distribution, a pattern observed in resistant embryos. Moreover, migration *mmp7*-expressing cells at the posterior end of the embryo after tail amputation correlates with the localization of foci of depolarized cells at the same site, suggesting that depolarization of host cells attracts migrating myeloid cells. Future studies will elucidate the precise mechanisms and signaling pathways involved in this phenomenon.

It is also possible that other mediators of innate immunity are impacted by bioelectricity: for example, antibacterial peptides secreted by the host’s cells or cell-autonomous defense (CAD) mechanisms. Interestingly, CAD involves membrane trafficking,^76, 77^ the function of which should be influenced by bioelectricity through modulation of intracellular membrane vesicles voltage potential.^78, 79^ Bladder epithelial cells, the mammalian targets of the bacterial strains used in our study, have been shown to react to infection through CAD mechanisms.^80^ It is also possible that the ontogeny rather than the function of immune cells is affected by bioelectric modulation: an acceleration of myelopoiesis could be responsible for the increase in migrating myeloid cells. The observed concentrated distributions of *spib-a* and *mmp7*-expressing cells in infected specimen could be indicative of macrophages’ impaired maturation. Future studies utilizing transgenics to drive tissue-specific overexpression of ion channels (targeting immune and other cell types) will help to pinpoint specific cell populations mediating the resistance to infection and response to bioelectric signaling.

### Signaling pathways involved in bioelectric modulation of infection resistance

We sought to elucidate which signaling pathways were used to modulate the innate immune response to bacterial infection. Since IVM was one of the most potent inducers of increased resistance to infection we identified, we focused our investigation on the downstream intracellular mediators of its effects. Previous studies have shown that IVM induces a hyper-activation of melanocytes (differentiated progeny of the neural crest) through transduction mechanisms involving serotonergic signaling and MSH action.^33, 34^ Moreover, immune cell types express genes coding for enzymes that participate in the synthesis and/or transport of serotonin.^81, 82^ When we treated embryos, chemically depolarized and infected with pathogenic *E. coli*, with specific inhibitors of serotonin transport, we observed that interfering with serotonergic signaling eliminated the increased resistance to infection induced by the depolarizing agents. These data implicate serotonergic signaling, which is consistent with previously shown roles of SERT-mediated serotonin signaling as a transducer of V_mem_ change to downstream cellular responses.^31, 34, 51, 55^

It is interesting to note that barium chloride treatment does not result in hyperpigmentation of the embryos, but blocking serotonergic signaling still nullifies its effect on resistance to infection. It is, therefore, likely that one or more serotonergic pathways distinct from the one leading to hyperpigmentation are involved in modulating the innate immune response. However, treatment with an MSH agonist, which acts downstream from the previously described serotonergic pathway involved in hyperpigmentation, leads to an increased resistance to infection equivalent to the one induced by IVM. It shows that this single pathway is sufficient to enhance innate immune response, but that distinct serotonergic pathways are involved when using a different depolarizing agent that does not lead to hyperpigmentation. Further studies aimed at elucidating the precise molecular mechanisms involved in these phenomena will likely yield a diversity of targets to be used as potential modulators of innate immune response. These studies may be especially important given recent data that SSRI exposure during embryogenesis affects immune response in mammals.^83^

### Role of melanocytes in innate immunity modulation

Previous studies have suggested that melanocytes could play an active role in fighting pathogens.^84, 85^ Our data support this hypothesis by showing that melanocyte-derived supernatants and cell lysates increase the resistance to infection in our amphibian model. Interestingly, only extracts derived from actively proliferating melanocytes induce this increase, while quiescent, melanin-secreting, and more differentiated melanocytes have no significant effect. This melanin-independent effect is supported by our observation that IVM, which leads to hyperpigmentation and increases resistance to infection, still increases the survival ratio of infected albino larvae, which have melanocytes but they do not secrete melanin. Moreover, we know that melanocytes migrate at wound sites following injury or amputation (Fig. 3a), which activates pathways common to the innate immune reaction. Some melanocytes then fragment, releasing factors that could enhance the effectiveness of the immune response, as supported by our data showing an increase in the resistance to infection following exposure to actively proliferating melanocytes. Further studies will be needed to conclusively distinguish the different contributions of melanocytes vs. other cell types, and identify melanocyte-derived factors induced by infection and/or enhancing the immune response.

### Intersection between resistance to infection and regeneration

**Fig. 7:**
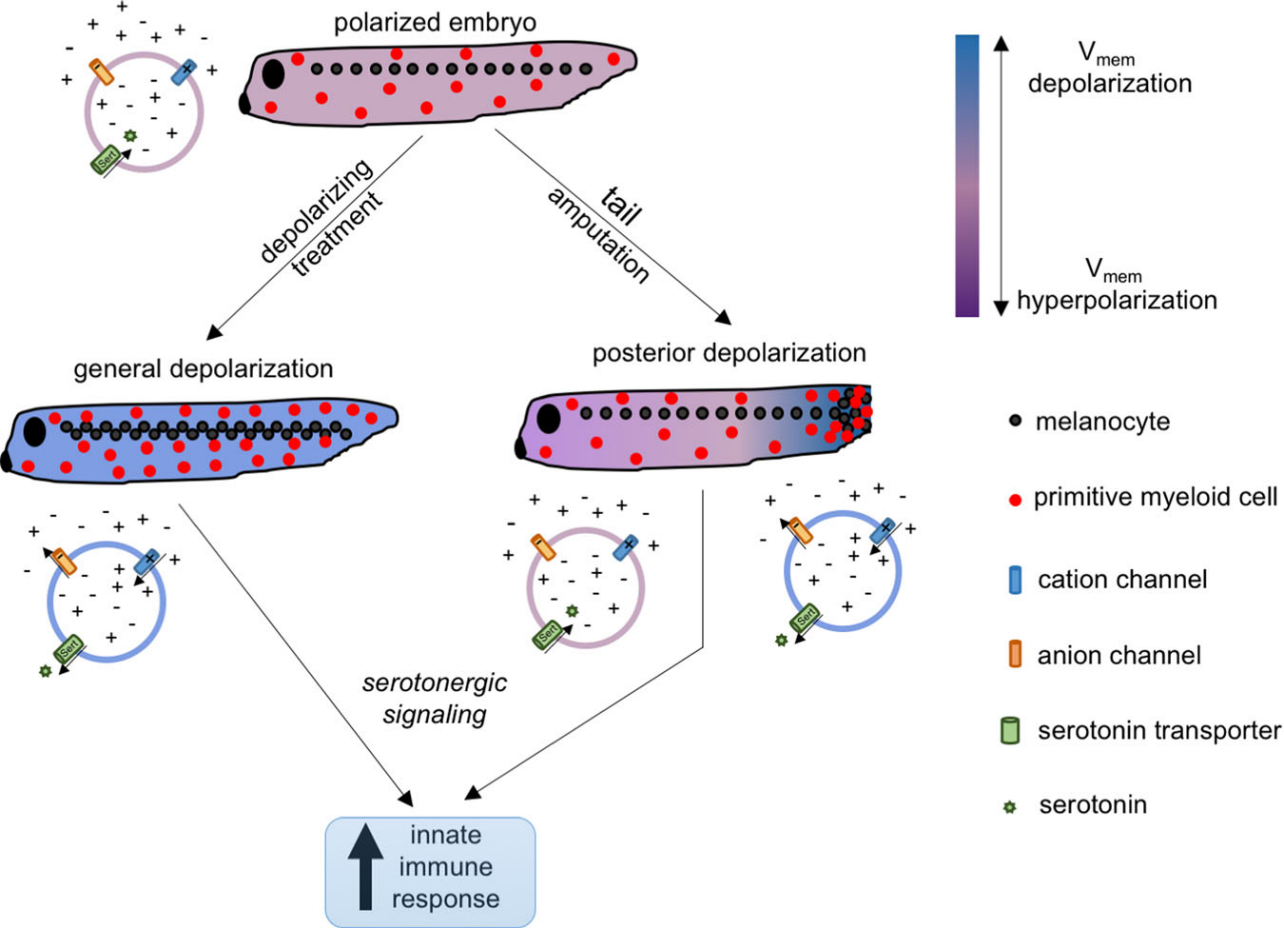
Model integrating *V*_mem_ signal into innate immune response. Unperturbed tadpoles harbor polarized cells, with specific native amounts and distributions of melanocytes and primitive myeloid cells. Following chemical/genetic treatments that depolarize *V*_mem_, pathways involving serotonin signaling induce proliferation and redistribution of melanocytes and primitive myeloid cells, leading to an increase in the efficiency of the immune response when stimulated with a pathogenic agent. Tail amputation induces a strong posterior *V*_mem_ depolarization (at the site of injury), where melanocytes and primitive myeloid cells are recruited, resulting in a net increase of the latter in the embryo, leading to an enhanced innate immune response

One of our most surprising results is the increase in survival to infection following tail amputation. Having an additional stress benefiting the resolution of a previous one is counterintuitive at first, since an accumulation of stresses could be expected to overwhelm the defense mechanisms of an organism. However, there is the possibility that having the organism exposed to two stresses each involving the induction of common defense mechanisms would enhance the efficacy of the response by increasing the probability of reaching the threshold necessary for eliminating the infectious agent. It is likely that the activation of immune cells by injury, via biochemical as well as bioelectrical signals triggered by regenerative response^86^ contributes to their ability to clear the body of infection. We suggest a model in which the host’s bioelectric status provides the bridge between regeneration and resistance to infection: posterior depolarization following tail amputation correlates with an increase in posterior migration of myeloid cells, suggesting that a more general depolarization of the host facilitates migration of these myeloid cells throughout the embryonic body and strengthens immune response to pathogenic invasion. Migration of melanocytes provides an additional layer of immune reinforcement through serotonergic pathways (Fig. 7). Another important connection between bacteria resident in the host and regeneration is through the production of butyrate, which is a known mediator of long-range bioelectric effects.^87–89^ Future studies will examine the bi-directional feedback between this and other compounds that may be produced by bacteria (and their modulation by the immune system) and the bioelectrically regulated aspects of regeneration.

The interplay between responses to physical injuries and infection has the potential to reveal new ways of treating both infections and severe physical injuries.

## Conclusion

We have identified bioelectricity and active regeneration as a new modulators of innate immunity. General depolarization and regeneration of the host leads to increased resistance to infection by uropathogenic *E. coli*, while its hyperpolarization shows the opposite effect. Bioelectric signaling in vivo can improve immune response by a number of effects on the ontogeny and activation of the innate immune system. These new mechanisms could be used to develop or enhance actual treatments for infectious diseases. Also, given the limited specificity of innate immunity, its enhancement will be important in the fight against emerging diseases faced by some populations, as well as against the probable synthesis of new pathogens. Moreover, since the innate immune response is crucial in initiating the adaptive response, bioelectricity could act as a variable that can be manipulated to enhance the effectiveness of vaccines. Finally, further exploring the role of bioelectricity in the immune response triggered by other pathogenic agents (for example, different bacterial species, viruses, and fungi) as well as non-pathogenic microorganisms will help us elucidate its breadth of impact in the immune response and the tolerance of commensal bacterial populations.

## Materials and methods

### Bacterial strains

All bacterial strains were generously provided by Dr. Matthew A. Mulvey (University of Utah, Salt Lake City, Utah, USA) and have been previously described.^25^ Briefly, we utilized a uropathogenic *Escherichia coli* strain (UTI89) and the K12 *E. coli* lab strain (MG1655) to elicit a pathogenic response. Both strains harbor a plasmid (pGEN-GFP(LVA)) encoding a mutated form of the GFP under the control of the constitutively active *em7* promoter. The LVA mutation destabilizes the GFP protein, resulting in a reduced half-life (around 40 min). Bacteria were grown at 37 °C in LB medium (MP Biomedicals, LLC) supplemented with 50 mg/mL ampicillin (Fisher). Exponentially growing bacteria were pelleted and resuspended in 1/10 volume in phosphate-buffered saline before injections in embryos.

### *Xenopus* husbandry and injection

*X. laevis* embryos were fertilized in vitro according to standard protocols^90, 91^ in 0.1X Marc’s modified Ringer’s (MMR) medium. All the experiments were approved by the Tufts University Animal Research Committee, protocol MR-2014-79. Embryos were incubated at 14 or 18 °C and staged according to Nieuwkoop and Faber.^92^ For mRNA injections, capped, synthetic mRNAs were generated using the mMessage Machine (Ambion) and injected into fertilized embryos at the one-cell stage. At the time of injection, embryos were kept in 3% Ficoll. Injections were performed using borosilicate glass needles calibrated for a bubble pressure of 55–60 kDa and using 150 msec pulses (delivering between 1 and 2 ng of mRNA). For infections, blastula or gastrula-stage embryos were injected with concentrated preparations of bacteria (see above) using borosilicate glass needles calibrated for a bubble pressure of 25–30 kDa and 150 msec pulses. Following infection, embryos were incubated at 21 °C. Even though bacteria were grown and concentrated following a constant methodology, and embryos were collected and grown under the same conditions, variation in survival rates was observed between experiments. This is likely due to differences in bacterial growth in situ, which can be affected by differences in the genetic background of each individual from different egg clutches. For these reasons, every individual comparison within an experiment was conducted using eggs from a single fertilization and a single suspension of bacteria. For each assay from each triplicate, at least 120 embryos were use for every treatments being compared.

### Drug exposures

Embryos were exposed to either depolarizing or hyperpolarizing compounds from Faber-Nieuwkoop stage 13/14. They were exposed in 0.1X MMR to one of the following compounds: 1 μM ivermectin (IVM) (Sigma), 0.2 mM BaCl_2_ (MP Biomedicals, LLC), 60 mM potassium gluconate (Sigma-Aldrich), 70 mM *N*-methyl-d-glucamine (NMDG) chloride, 10 μM fluoxetine (Sigma-Aldrich), or 500 nM SHU9119 (Tocris).

### Immunofluorescence and microscopy

Spatial detection of leukocytes was performed by immunofluorescence with the XL2 antibody^52^ on whole embryos. Embryos were fixed 1 h in MEMFA^90, 91^ and washed three times in phosphate-buffered saline (PBS). They were then permeabilized in PBS supplemented with 2% bovine serum albumin (BSA) and 0.1% Triton X-100 for 30 min at room temperature. It was followed by a one-hour blocking step at room temperature in PBT (PBS + 2% BSA + 0.1% Tween-20) supplemented with 10% heat-inactivated goat serum. The embryos were then incubated overnight at 4 °C with the primary antibody (monoclonal mouse anti-XL2) diluted 1:500 in blocking buffer. On the next day, they were washed six times (1 h each time) at room temperature in PBT, before being blocked for 30 min and incubated overnight with the secondary antibody (goat anti-mouse IgG conjugated with Alexa-Fluor 555 (Invitrogen)) at 4 °C. The following day, animals were washed six times in PBT and photographed using a Nikon SMZ1500 microscope equipped with a Hamamatsu ORCA AG CCD camera and controlled with the QCapture software.

### GFP activity quantification

To quantify bacteria-derived GFP activity in whole-embryo lysates, we adapted a protocol developed to quantify GFP activity in sea urchin embryos.^93^ Briefly, embryos were harvested individually and suspended in 50 μL of ice-cold lysis buffer (1.5 mg/mL BSA, 50 mM Tris-HCl (pH 8.0), 150 mM NaCl, 10% glycerol, 0.5 mM EGTA, 0.1% Triton X-100, and 0.1% Nonidet P-40). They were stored at −20 °C from one to 7 days before being mechanically sheared with a pipet tip and centrifuged at 14,000 rpm at 4 °C for 30 min. Thirty microliters of the supernatant were transferred into a well on a black-bottom 96-well plate and the GFP activity was measured using a SpectraMax M2 (Molecular Devices) spectrofluorometer with an excitation wavelength of 475 nm and an emission wavelength of 515 nm.

### Cell culture and exposure to bacteria

Human neonatal primary melanocytes (HEMn, Lifeline Technologies) were cultured in DermaLife M melanocyte medium (Lifeline Technologies) supplemented with 100 U/mL penicillin/streptomycin (Invitrogen). For supernatant harvesting, dishes at 50% confluency (for proliferating HEMn condition), or confluent for 2 days (for quiescent HEMn condition), were refreshed and cultured for 24 h in DermaLife M medium, after which the medium was harvested and stored at −20 °C until used for exposure to bacteria. For non-denaturing cell lysates, 10-cm dishes at 80% confluency or confluent were scraped and cells pelleted and snap frozen. The pellets were then resupended in 1 mL lysis buffer (1.5 mg/mL BSA, 50 mM Tris-Cl pH 8.0, 150 mM NaCl, 10% glycerol, 0.5 mM EGTA, 0.1% Triton-X-100, 0.1% Nonidet P-40) for 1 h, spun for 30 min at 14,000 rpm, and aliquoted and stored at −20 °C. For bacterial exposures to cell supernatants or lysates, bacteria were processed as previously described but, instead of being resuspended in phosphate-buffered saline, they were resuspended in an equivalent volume of supernatant or lysate. The bacterial suspension was kept at room temperature for 30 min prior to infection.

### In situ hybridization

In situ hybridization was performed as previously described.^90, 91^ Specimens were washed in 0.1% Tween-20/PBS (PBS-T) and dehydrated through increasing concentrations of methanol. Probes were generated in vitro from linearized templates using a dioxygenin (DIG)-labeling mix. Probes used were *Xenopus laevis spib-a* (GE Dharmacon) and *Xenopus tropicalis mmp7*.

### Voltage profiling in vivo

Embryos were transferred to a medium containing 0.95 μM of the fluorescent voltage reporter dye DiBAC_4_(3) (refs. 94, 95) for 30 min. Imaging was performed using a Nikon SMZ1500 microscope equipped with a Hamamatsu ORCA AG CCD camera and controlled with the QCapture software. Fluorescence levels were then quantified using ImageJ.

### Tail amputation assay

Embryosat NF stage 27 were anesthetized with 0.02% tricaine methane sulfonate (MS222) solution; tail buds were amputated from the most posterior fifth portion of their body using a scalpel blade. Non-amputated embryos were also incubated in the same amount of tricaine for an equivalent time.

### Statistical analyses

Statistical analyses were performed using Microsoft Excel^TM^. Data was either pooled from various iterations, with *χ* ^2^-Square analysis performed on them, or data from various iterations were analyzed by *t-*test (for two groups) or analysis of variance (for more than two groups).

### Transcriptome analysis

To determine whether depolarization events were associated with the expression of genes associated with the innate immune system, two recent expression studies in *X. laevis* were leveraged and reanalyzed.^33, 40^ Detailed methods for each experiment can be found in each corresponding manuscript. Briefly, embryos were exposed to 1 µM IVM (Sigma) or microinjected in 3% Ficoll^91^ with capped synthetic mRNAs (mMessage mMachine kit, Ambion) encoding depolarizing and hyperpolarizing ion channels at the 4-cell stage into the middle of the cell in the animal pole. The embryos were analyzed at stage 45 via microarray analysis to identify transcripts that were up or downregulated due to the induced *V*_mem_ changes.

Microarray hybridizations were performed by the Beth Israel Deaconess Medical Center (BIDMC) Genomics core (Boston, MA) at Harvard University. Microarray hybridization was performed using the Affy 3’ IVT Express Kit (Affymetrix, Santa Clara, CA) as per the manufacturer’s protocol. Fragmented and biotin labeled/amplified RNA was hybridized in the GeneChip *Xenopus laevis* Genome 2.0 array (Affymetrix, Santa Clara, CA) as per the protocol provided by the manufacturer. The Affymetrix GeneChip® *X. laevis* Genome 2.0 Array has 32,400 probe sets representing more than 29,900 *X. laevis* transcripts. The quality of hybridized arrays was assessed using Affymetrix guidelines on the basis of scaling factor, background value, mean intensity of chip and 3’ to 5’ ratios for spike-in control transcripts. Other aspects of this analysis have been reported previously.^17, 33^ All data have been deposited to the National Center for Biotechnology Information Gene Expression Omnibus database (accession no. GSE70834, platform GPL10756).

### Bioinformatics

Sub-network enrichment analysis (SNEA) was performed in Pathway Studio 10.0 (Elsevier Life Science Solutions) and ResNet 10.0 for constructing gene interaction network for transcripts showing differential expression. Differentially expressed genes^33, 40^ were mapped onto disease networks using official gene symbols (Name + Alias). SNEA was performed and significantly enriched processes were determined to be those with *P* < 0.05 that also contained more than five members in the network. These were the networks that are most represented by the entities in each gene list. Venn diagrams^96^ were generated using gene symbols to identify common genes between IVM depolarization^33^ and all proteins that had a direct connection to innate immunity in Resnet 10.0. Networks are constructed based on expression, binding, and regulatory interactions using direct connections with one neighbor. Sub-networks that included “expression targets”, and “disease” were the focus of these investigations.

## Electronic supplementary material

Supplementary Information

Supplementary Figure 1

Supplementary Figure 2

Supplementary Figure 3

Supplementary Figure 4

Supplementary Figure 5

Appendix 1

Appendix 2
