# Supplementary material for: Bioelectric regulation of innate immune system function in regenerating and intact Xenopus laevis
Source: NPJ Regen Med. 2017 May 26;2:15. doi: 10.1038/s41536-017-0019-y (PMC5677984; doi:10.1038/s41536-017-0019-y)
Supplement: Supplementary file 1 — Supplementary Information [file 41536_2017_19_MOESM1_ESM.docx]

Bioelectric Regulation of Innate Immune System Function

in Regenerating and Intact *Xenopus laevis*

**Supplementary Figure Legends**

**Supplementary Figure 1.** Embryos treated with barium chloride are more depolarized. Intensity of DiBAC_4_(3) signal in non-infected medium vs barium chloride-supplemented medium (0.2 mM).

**Supplementary Figure 2.** Potassium gluconate is the only chemical compound used in our depolarization / hyperpolarization experiments that significantly impacts uropathogenic bacterial growth. The specified compounds were added directly in LB broth at the same concentrations used on embryos (1 μM ivermectin, 0.2 mM BaCl_2_, 60 mM potassium gluconate, and 70 mM N-methyl-D-glucamine (NMDG) chloride). 50 μL of an overnight uropathogenic *E. coli* culture were added to 4 mL LB broth supplemented or not with a depolarizing or hyperpolarizing compound. 1 mL from these culture were harvested at 0, 2 and 4 hours of culture, from which bacteria were pelleted and submitted to non-denaturing lysis in a protocol identical to the one previously used for embryo lysis. GFP activity was quantified by spectrofluoremetry.

**Supplementary Figure 3.** Surviving *X. laevis* embryos show peripheral mobilization of leukocytes following injection of uropathogenic *E. coli* at gastrula stage. A) Fluorescence detection of leukocytes with the specific XL2 antibody in embryos surviving four days after infection. Panel i shows a non-infected embryo while panel ii shows an infected one. The white polygon shows the post-anal ventral fin area used to calculate the concentration of peripheral leukocytes. B) Comparative concentrations of peripheral leukocytes calculated from the post-anal portion of the ventral fin (delimited by the white polygons in figure 3A). The number of red fluorescent was calculated using ImageJ and relative concentrations compared with control non-infected embryos independently for each set. Panel i, chemical depolarization; panel ii, genetic depolarization; panel iii, chemical hyperpolarization; panel iv, genetic hyperpolarization. Error bars represent the standard deviation from at least three individual embryos. * denotes p<0.05 when compare to non-infected embryos in the corresponding treatment.

**Supplementary Figure 4.** Quantification of uropathogenic *E. coli*-derived green fluorescence using spectrofluorometry on native embryo lysates three days after infection. The graph shows the first quartile (bottom of orange rectangles), median (intersection of orange and grey rectangles), and the third quartile (top of grey rectangles) values for each chemical polarization conditions. The thick black lines represent the average values. Asterisks indicate statistically significant difference in the distribution of GFP fluorescence intensity when compared to control condition (p=0.019 and 0.008 for potassium gluconate and NMDG-chloride, respectively; ANOVA two-way test).

**Supplementary Figure 5.** Detectable levels of bacteria-derived GFP expression 48 hours after infection indicate future lethality. 24 hours after infection, bacterias concentrate in the extra-embryonic space inside the vitelline membrane (panels i-ii) and, after hatching, most embryos do not show detectable levels of bacteria-derived GFP activity in areas other than the released vitelline membrane (panels iii-iv). 48 hours after infection, embryos that show detectable levels of bacteria-derived GFP activity will succumb to infection within the following two days while the embryos from which no GFP activity can be detected will survive (resistant). Fluorescent imaging in panels i, ii, iii; bright field imaging in panels ii, iv, vi.
