# Supplementary figures and images for: Bioelectric regulation of innate immune system function in regenerating and intact Xenopus laevis
Source: NPJ Regen Med. 2017 May 26;2:15. doi: 10.1038/s41536-017-0019-y (PMC5677984; doi:10.1038/s41536-017-0019-y)

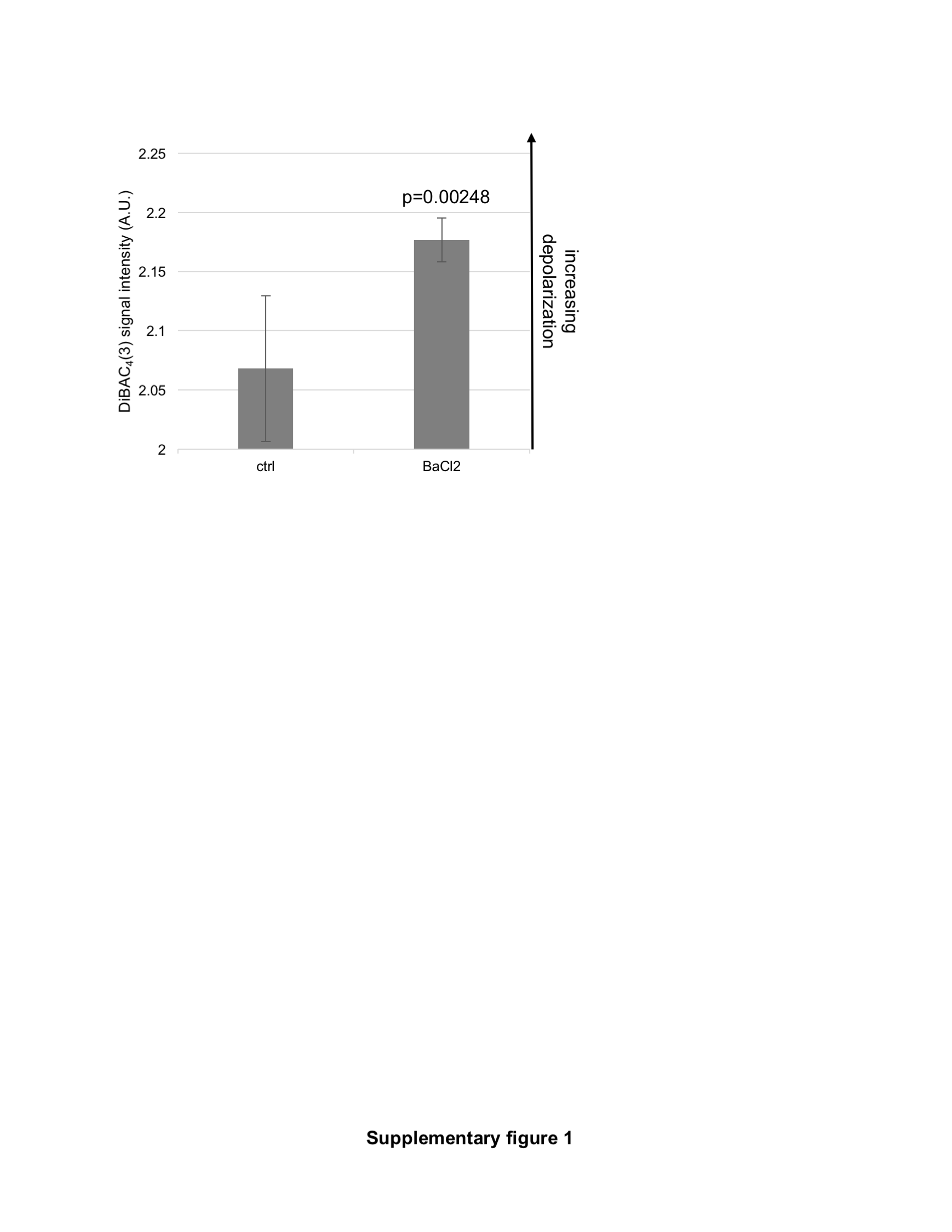

Supplement: Supplementary file 2 — Supplementary Figure 1 [file 41536_2017_19_MOESM2_ESM.tif]

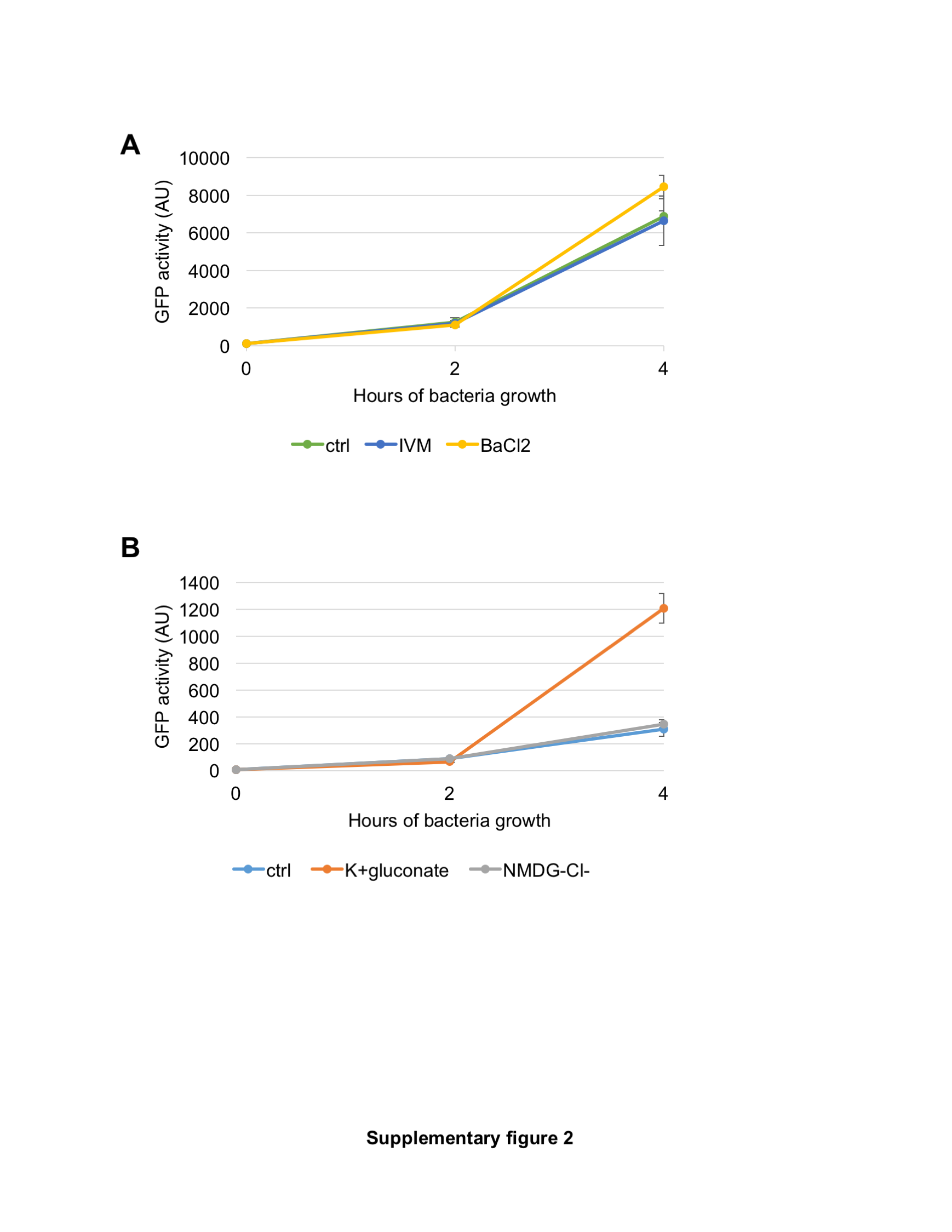

Supplement: Supplementary file 3 — Supplementary Figure 2 [file 41536_2017_19_MOESM3_ESM.tif]

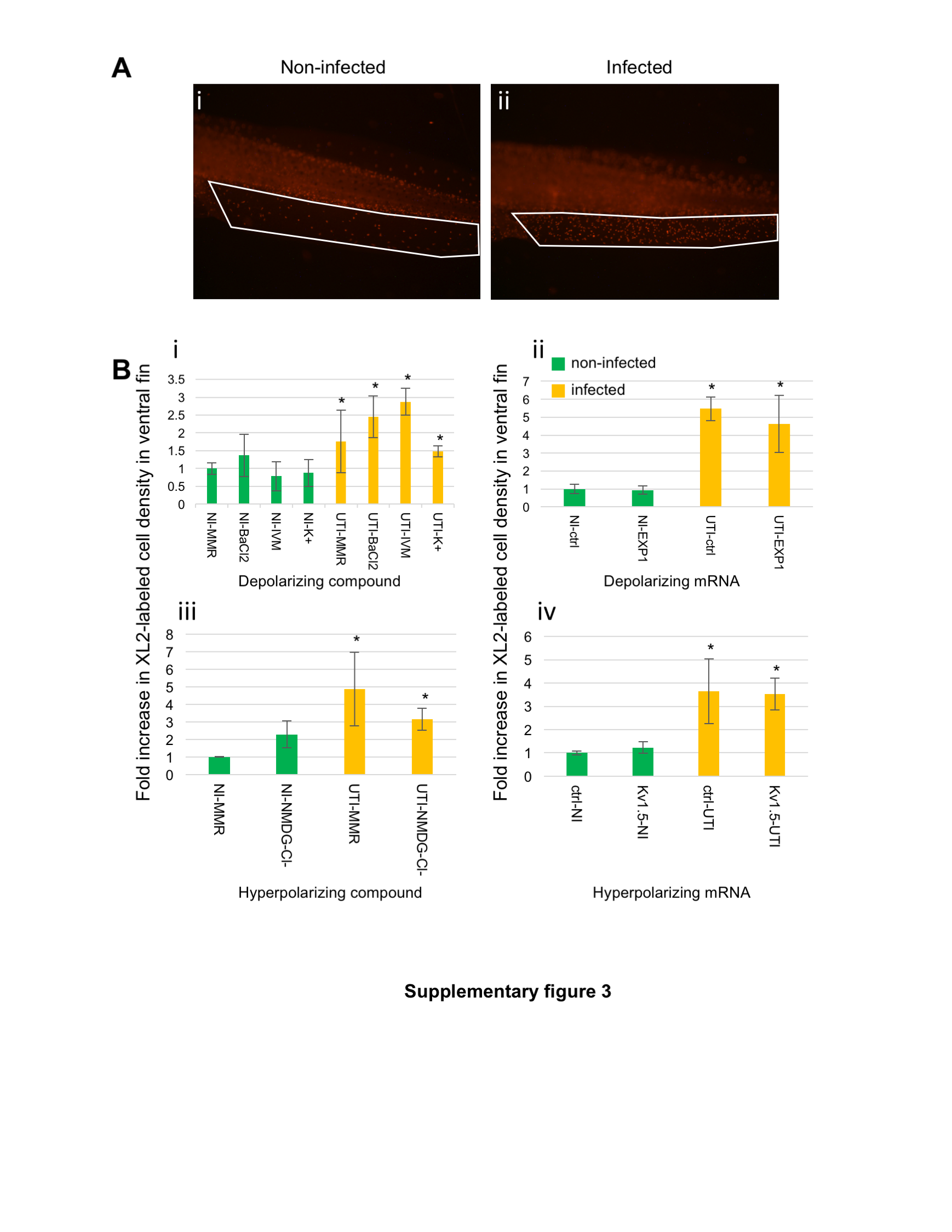

Supplement: Supplementary file 4 — Supplementary Figure 3 [file 41536_2017_19_MOESM4_ESM.tif]

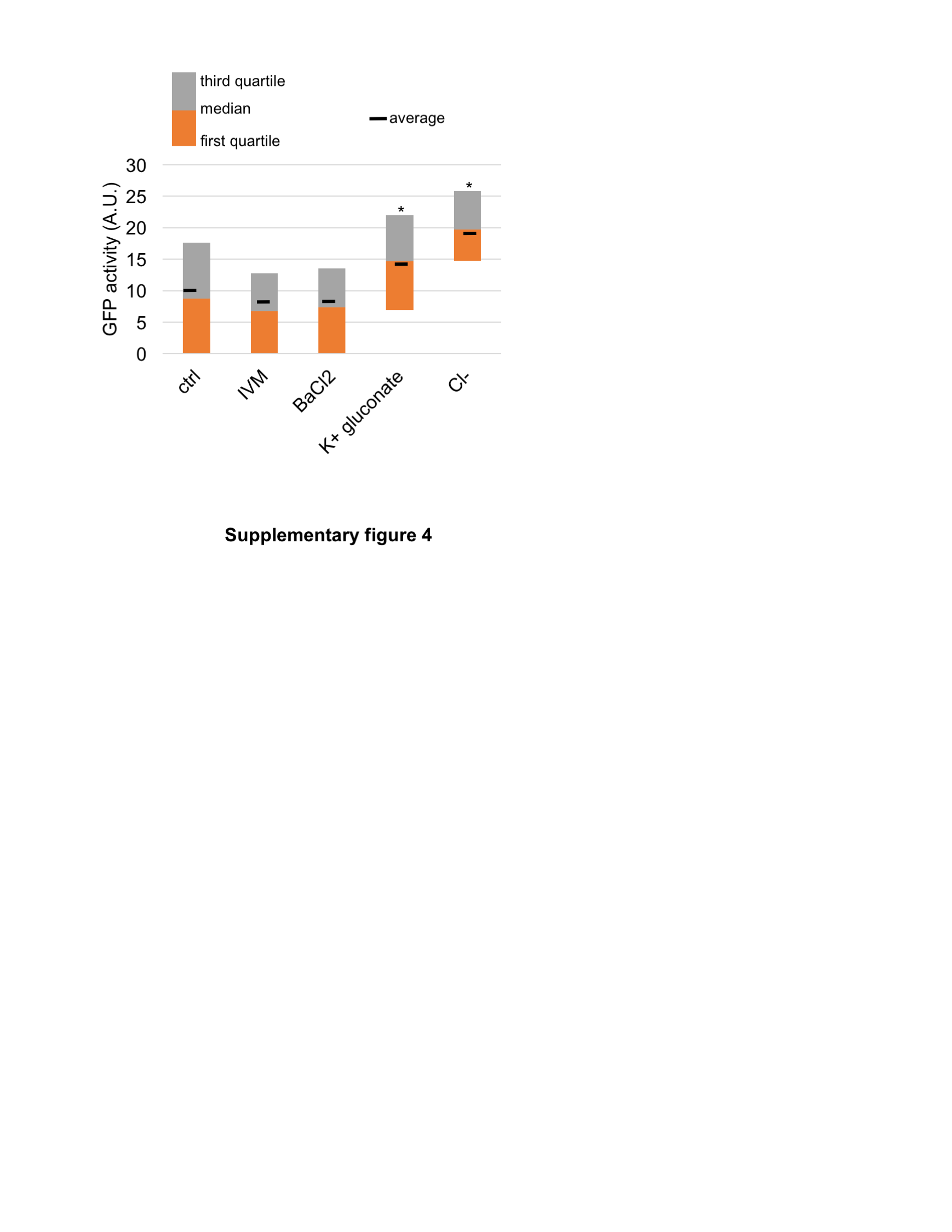

Supplement: Supplementary file 5 — Supplementary Figure 4 [file 41536_2017_19_MOESM5_ESM.tif]

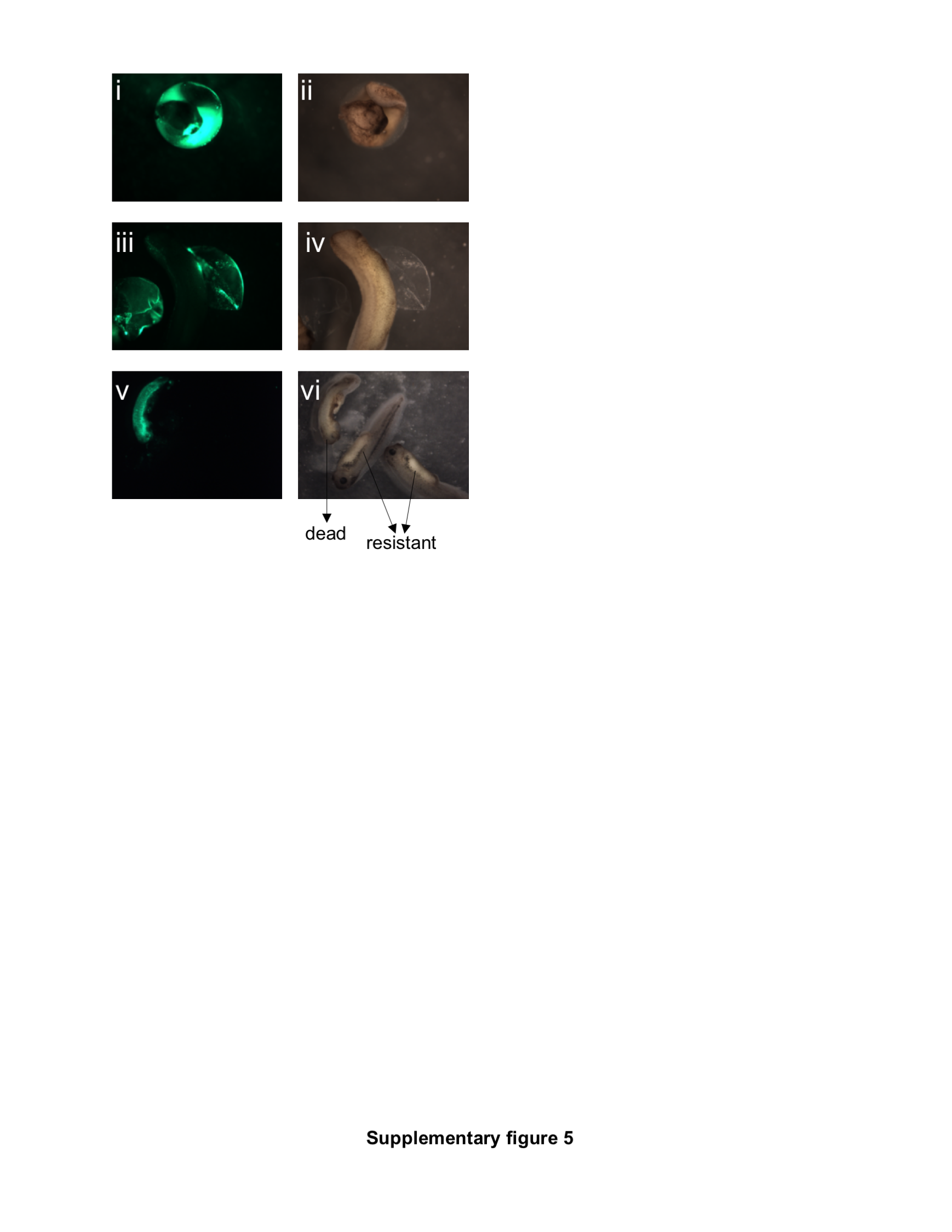

Supplement: Supplementary file 6 — Supplementary Figure 5 [file 41536_2017_19_MOESM6_ESM.tif]
